# Combined metabolomics and 16S rDNA sequence analyses of the gut microbiome reveal the action mechanism of Fructus Akebiae against hepatic fibrosis

**DOI:** 10.3389/fmed.2024.1492383

**Published:** 2025-02-05

**Authors:** Rong-Rong Wu, Duo-Rui Nie, Fang-Hui He, Zhi-Hang Li, Fei Xu

**Affiliations:** ^1^School of Pharmacy, Hunan University of Chinese Medicine, Changsha, Hunan, China; ^2^Graduate School, Hunan University of Chinese Medicine, Changsha, China; ^3^College of Pharmacy, Hunan University of Chinese Medicine, Changsha, China; ^4^Hunan Engineering Technology Research Center for Bioactive Substance Discovery of Chinese Medicine, Changsha, China; ^5^Hunan Province Sino-US International Joint Research Center for Therapeutic Drugs of Senile Degenerative Diseases, Changsha, China

**Keywords:** Fructus Akebiae, hepatic fibrosis, network pharmacology, 16s rDNA sequencing, *Akkermansia*, *Verrucomicrobiota*

## Abstract

**Objectives:**

To explore the mechanism underlying the effect of Fructus Akebiae (FAE) against hepatic fibrosis in mice through combined network pharmacology, liver metabolomics, and 16S rDNA analyses of the gut microbiota.

**Methods:**

In this study, we randomly divided mice into the control, model, FAE high-dose, FAE medium-dose, and FAE low-dose groups to analyze the pathological changes in the hepatic fibrosis and levels of the *α*-SMA, collagen 1, Nuclear Factor Kappa B (NF-*κ* B), Toll Like Receptor 4 (TLR4). The gut microbiota was analyzed through 16S rDNA sequencing analysis of liver metabolites using liquid chromatography-mass spectrometry. Furthermore, network pharmacology was used to determine the specific molecular regulation mechanism of FAE in hepatic fibrosis treatment.

**Results:**

FAE treatment markedly improved the pathological changes in the hepatic fibrosis. Analysis revealed that FAE administration reversed the carbon tetrachloride (CCl_4_)-induced dysbiosis by increasing the abundance of *Akkermansia* and reducing that of *Cyanobacteria*. Additionally, metabolomic analysis showed that FAE treatment reversed the CCl_4_-induced metabolic disorders by regulating amino and nucleotide sugar metabolism. Furthermore, correlation analysis showed that *Akkermansia* and *Verrucomicobiota* were closely related to D-tolasaccharide and maltotetraose saccharide. Moreover, network pharmacology indicated that FAE might regulate the signaling pathway through the JUN/CASP3/NOS3/PTGS2/HSP90AA1 during treatment.

**Conclusion:**

FAE may be a promising treatment for hepatic fibrosis, and its protective effects are associated with improvements in the microbiome and metabolic disorders.

## 1 Introduction

Hepatic fibrosis (HF) is a pathophysiological process characterized by the abnormal proliferation of connective tissue in the liver caused by injury through exposure to toxic chemicals, chronic hepatitis viral infection, autoimmune liver disease, and alcoholism (1, 2). HF leads to structural disorders of the liver, nodular regeneration of hepatocytes, and liver cirrhosis, which can, in turn, lead to fatal liver failure (3). The only available curative treatment option for patients with advanced cirrhosis is liver transplantation; therefore, early control of hepatitis and HF development has become the focus of clinical treatment (4).

The liver is closely connected to the intestine; therefore, the establishment of a homeostatic environment of mutualistic symbiosis between intestinal microorganisms and the organism is crucial for maintaining liver health (5). Damage to the intestinal barrier, inflammatory reactions, and metabolic abnormalities resulting from an imbalance in the intestinal flora and their metabolites may be critical factors in the development of HF (6–8). An imbalance in the intestinal flora often results in the reduction of the relative abundance of intestinal probiotic bacteria and intestinal barrier dysfunction, thereby accelerating the progression of HF. Hence, maintaining the homeostasis of intestinal flora can influence and potentially reverse the development of HF (9). Metabolomics is an experimental technique used for a comprehensive quantitative analysis of the changes in endogenous metabolites (10). Differential metabolite analysis and the identification of key biomarkers and associated metabolic pathways allow the examination of metabolic differences among various sample groups, offering new insights into disease mechanisms (2, 7, 11). The holistic and systematic characteristics of network pharmacology (NP) align with the holistic view and evidence-based treatment approach of Chinese medicine, offering a promising framework for integrating preclinical research from both Chinese and western medicine perspectives (12).

In recent years, significant advancements in Chinese herbal medicine have contributed to the prevention of HF (13, 14). FAE is derived from the dried, nearly mature fruits of *Mucuna pruriens*, *Mucuna trilobata*, and *Mucuna alba*, all of which belong to the Mucunaceae family (15). It is characterized by its bitter taste and cold nature and is associated with the liver, gallbladder, stomach, and bladder meridians. FAE is known for its ability to strengthen the spleen, regulate the Qi, remove blood stasis, soften hard masses, eliminate excess heat, and detoxify. FAE has both anti-HF and hepatoprotective effects (16). When combined with contraindicated drugs, the side effects of FAE mainly include gastrointestinal discomfort, drug allergies, increased risk of bleeding, impact on blood sugar and blood pressure, drug interactions, etc., but it is not toxic (17). However, the mechanism whereby FAE resists HF, as illustrated by the analysis of the alteration in metabolites and species composition of the intestinal flora, remains unclear.

Therefore, in this study, we aimed to analyze the changes in metabolic pathways and the abundance of beneficial and harmful flora in the cecum intestinal flora of mice with CCl_4_-induced HF treated with FAE, using metabolomics and 16S rDNA sequencing, and investigate the interaction between the core active components of FAE and key targets during HF treatment through molecular docking and NP. By predicting the targets and pathways underlying the efficacy of FAE in treating HF, we sought to elucidate its protective effects on HF mice by examining microbial changes and provide a theoretical foundation for its future development and utilization.

## 2 Materials and methods

### 2.1 Materials and reagents

We procured male 8 weeks BALB/c mice (body weight: 18–22 g,) from Hunan SJA Laboratory Animal Co., Ltd. (LL20240312001). All the animals were raised in Specific Pathogen Free level barrier facilities at the Animal Experiment Center of Hunan University of Traditional Chinese Medicine (located in Changsha, China) which maintain a ventilated living environment, suitable humidity and temperature, sufficient food and drinking water. All the experimental procedures were approved by the Animal Ethics Committee.

We randomly divided 30 mice into the following five groups: control, model, high-dose FAE, medium-dose FAE, and a low-dose FAE groups, with six mice in each group. Except for the blank group, all other groups were injected with 0.2 mL of 20% CCl_4_ (Fuchen Chemical Reagent Co., Ltd., Tian-Jing, China) (CCl_4_ and olive oil in a 1:4 ratio) twice a week for a total of 4 weeks. The blank group was administered with the same amount of ordinary olive oil.

FAE (0.2 mL; Shanghai Aladdin Chemical Co., Ltd., Shanghai, China) was administered to mice through gavage (at concentrations ranging from 0.125 to 1.25 g/mL) once a day for 4 weeks. According to the Chinese Pharmacopoeia, the clinically used dose of FAE is 3–9 grams(g). According to the Chinese Pharmacopoeia, the clinically used dose of FAE is 3–9 grams (17). Therefore, a pre-test was conducted with reference to this dose range, and it was finally decided to use 0.125 g/mL as the low dose group, 0.5 g/mL as the medium dose group, and 1.25 g/mL as the high dose group. The mice in both the blank and model groups were administered normal saline for 4 weeks following the same administration regimen as for the FAE groups. The drug intervention period for all groups commenced from the first week of modeling.

At the end of the experiment, all animals underwent fasting for 24 h. Blood, liver, and intestinal contents were then sampled. Blood (0.5 mL) was collected from the orbital vein. The serum was obtained through centrifugation; the remaining serum and intestinal contents were stored in a refrigerator at −80°C for further testing. Liver specimens were weighed and stored in paraformaldehyde.

### 2.2 Liver index

The mouse liver was removed, rinsed with phosphate-buffered saline, and the water was eliminated. The wet weight was measured as follows: Liver index = liver weight (g)/mouse body mass (g) × 100%.

### 2.3 Liver histopathology

Liver tissues were fixed in 4% paraformaldehyde and embedded in paraffin. Liver tissue was collected from 3 μm-thick sections. Hematoxylin and eosin (H&E) staining (Wuhan Weier Biotechnology Co., Ltd., Wuhan, China) was performed for pathological evaluation. Masson’s trichrome staining was performed to assess collagen content.

### 2.4 Serum biochemistry

Blood was allowed to stand at room temperature for 2 h and then centrifuged at 3,000 rpm for 10 min to obtain the serum. The serum levels of alanine aminotransferase (ALT), aspartate aminotransferase (AST), triglyceride (TG), and total bilirubin (T-Bil) were determined using kits from Shenzhen Mindray Biomedical Electronics Co., Ltd., Shen-Zhen, China, following the manufacturer’s instructions.

### 2.5 Immunohistochemistry

Paraffin-embedded liver tissues were cut into 3 cm-thick sections. After deparaffinization, antigen recovery was performed, followed by an overnight incubation at 4°C with rabbit anti-*α*-SMA (1:600), anti-collagen 1 (1:600), anti-TLR4 (1:500), anti-NF-*κ* B (1:500), anti-JUN (1:1000), anti-HSP90A11 (1:500), anti-PTGS2 (1:500), and anti-CASP3 (1:600) antibodies obtained from Anjie High Tech Wuhan Weier Biotechnology Co., Ltd. Hematoxylin was used for nuclear counterstaining, and a light microscope was used to capture TIFF images. They were quantitatively analyzed by Case Viewer (2.4_RTM_v2.4.0.)

### 2.6 Liver metabolomics

The Ultimate 3,000 ultra high-performance liquid chromatograph and Thermo Q-Exactive Orbitrap mass spectrometer were used for ultrapure LC–MS/MS analysis. Chromatographic separations were carried out on a Waters ACQUITY UPLC HSS T3 column (100 mm × 2.1 mm × 1.8 *μ* m). Subsequently, all raw data were analyzed using Progenesis QI and SIMCA-P14.0 software. Partial least-squares discriminant analysis (PLS-DA) and orthogonal partial least-squares discriminant analysis (OPLS-DA) were performed to identify overall metabolic differences among the three groups, with variable importance in the projection (VIP) used to identify characteristic metabolites. Distinct metabolites were analyzed based on VIP > 1.0 and *p* < 0.05. Additional information is provided in Supplementary materials.

### 2.7 Fecal DNA extraction and high-throughput 16S rDNA sequencing

16S rDNA gene sequencing of fecal samples was conducted. The samples were subjected to polymerase chain reaction (PCR) amplification, followed by amplicon purification and paired-end sequencing to evaluate the raw data.

### 2.8 Gut microbiota–metabolite correlation analysis

Differential metabolites involved in important metabolic pathways in the control, model, and FAE groups were identified using correlation analysis; 45 most abundant liver species across all the three groups were identified. Spearman correlation analysis and heatmaps were used to assess the correlation between the gut microbiota and metabolite content.

### 2.9 NP analysis

FAE targets were predicted using TCMSP,^1^ and disease targets were obtained from the Gene Cards database. Using “Hepatic fibrosis” as the search term and limiting the species to “*Homo sapiens*,” predict the relevant targets of HF in the Gene Card database, and obtain targets with AS related scores greater than 1.0 as the relevant targets of HF. On the Venny 2.1.0 drawing tool platform^2^ take the intersection of precognitive sub targets and HF related targets, obtain the intersection targets of precognitive active ingredients and HF, and draw a Venny plot. A protein–protein interaction (PPI) network was assembled using the STRING database version,^3^ with the organism set to *Homo sapiens*; only those interactions with a minimum score of >0.4 were considered significant. Gene Ontology (GO) and Kyoto Encyclopedia of Genes and Genomes (KEGG) pathway enrichment analyses were performed to further explore the biological functions of the targets and related pathways. To validate the binding of core components in FAE to the predicted core targets, the 3D molecular structures of the compounds were retrieved from the PubChem database, and the structural files of the target proteins were obtained from the RCSB Protein Data Bank.^4^ Molecular docking calculations were performed using SYBYL-X 2.0.

### 2.10 Statistical analysis

All data are presented as the mean ± SD of values from at least three independent experiments. Statistical analysis and comparisons between groups were performed using Prism 9.0 software (GraphPad Software, United States) and a one-way ANOVA. Statistical significance was set at *p* < 0.05.

## 3 Results

### 3.1 FAE attenuates HF in mice

**Figure 1:**
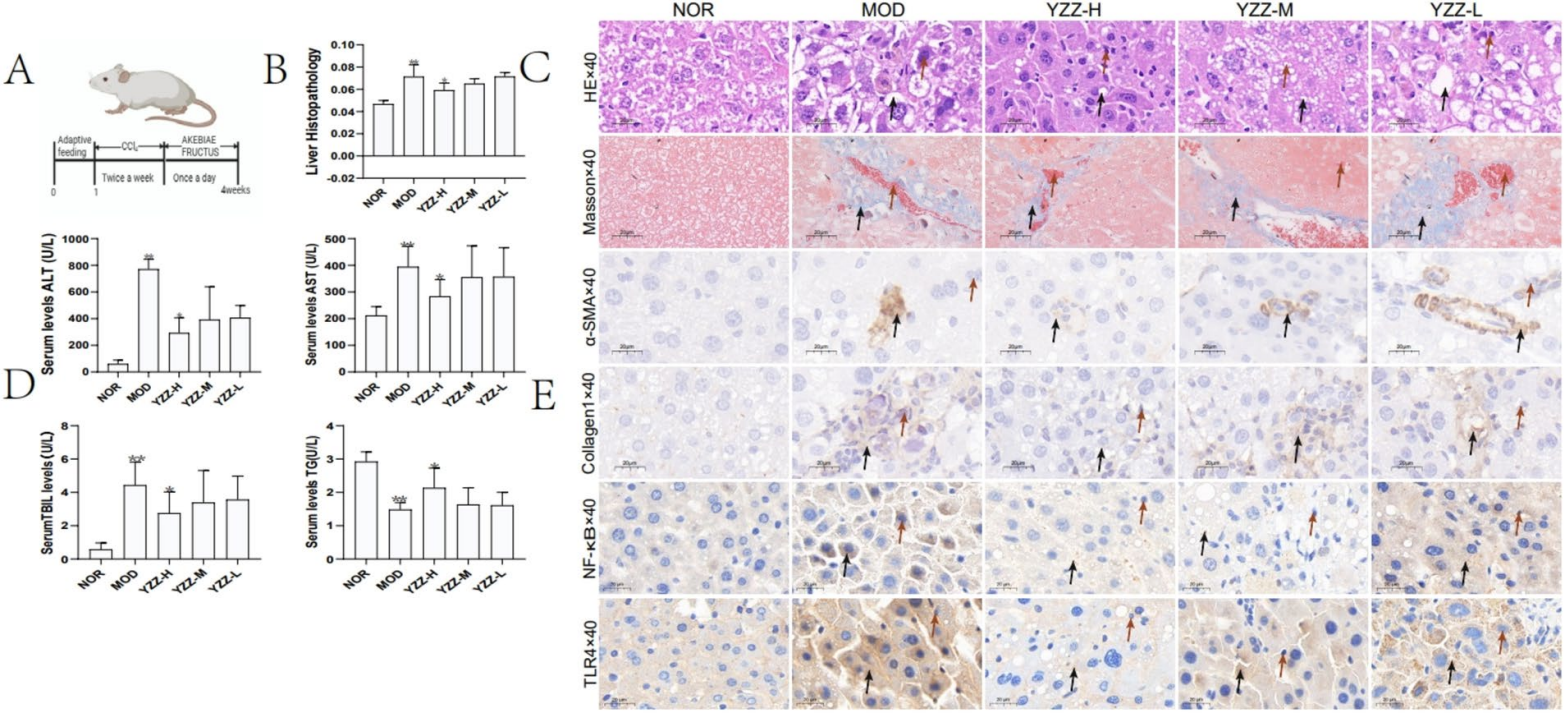
Effect of FAE on pathological changes of liver tissue. **(A)** Preparation of the hepatic fibrosis model and the drug intervention process. **(B)** Liver index. **(C)** HE and Masson staining. **(D)** Liver function via AST, ALT, T-Bil and TG expression. **(E)** Immunohistochemistry. *n* = 8, ** *p* ≤ 0.05 versus NOR; * *p* ≤ 0.05 versus MOD. C: →: necrocytosis; HE staining→: fat vacuole; Masson staining→: collagen deposition, E: →: necrocytosis; →: the express of *α*-SMA, Collagen1, NF-κB and TLR4, BD: *p* ≤ 0.05 represents a significant change. NOR, control group; MOD, model group; YZZ-H, high-dose FAE group; YZZ-M, medium-dose FAE group; YZZ-L, low-dose FAE group.

We examined the *in vivo* effects of FAE in male mice with CCl_4_-induced HF (Figure 1A). H&E staining results showed that the hepatic lobule structure of the mice in the blank group was complete, the hepatocytes were neatly arranged, the central vein was radial, and no infiltration of inflammatory cells into the portal area had occurred. Inflammatory cell infiltration was observed in the portal areas in the model group. A small number of inflammatory cells infiltrated the portal area of the high-dose FAE group, the fibrous septum was reduced, and the pathological changes were less severe than those in the model group. Masson staining showed that the blank group had intact liver tissue, no collagen deposition, and hyperfibrous tissue; in the model group, a large amount of collagen deposition occurred in the central vein and portal area, the hyperfibrous tissue formed fibrous cables connecting the adjacent collecting areas to form false lobules, and the degree of collagen deposition in the FAE high-dose group was significantly reduced. In summary, FAE treatment reduced HF (Figure 1C). These results were based on the liver index (Figure 1B) and serum AST, ALT, TG, and T-Bil levels (Figure 1D).

### 3.2 FAE affects the activities of *α*-SMA, collagen 1, NF-*κ* B, and TLR4

Given that α-SMA and collagen 1 expression by activated type HSC specifically in the liver can play a crucial role in HF progression. NF-κ B and TLR4 can upregulate the transcription and translation of pro-inflammatory genes and further promoting HF (18), inhibiting their expression could potentially mitigate fibrosis. Immunohistochemistry experiments revealed that FAE treatment suppressed the expression of α-SMA, collagen 1, NF-κ B and TLR4 (Figure 1E).

### 3.3 Effect of FAE on the metabolites of mice with CCl_4_-induced HF

**Figure 2:**
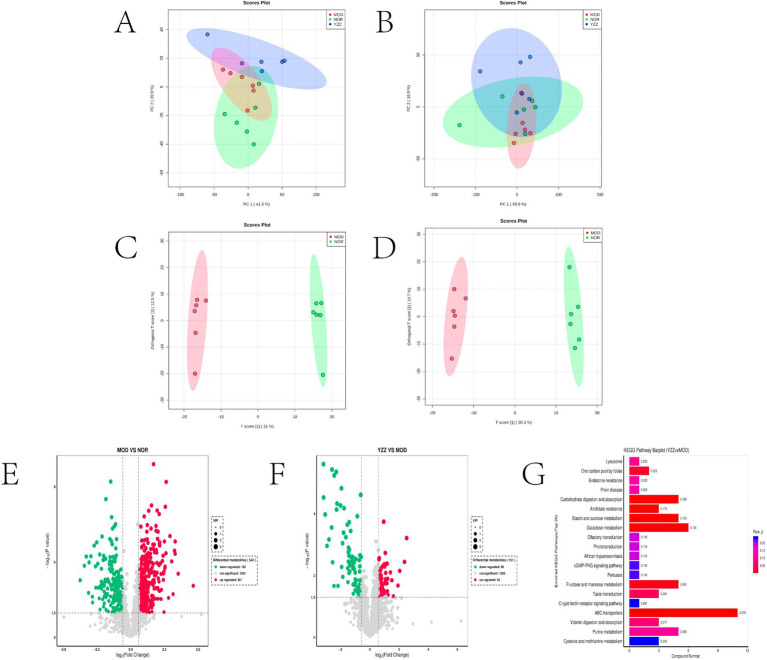
Effect of FAE on metabolites of hepatic fibrosis in mice. **(A)** PCA of quality control samples in positive ion mode. **(B)** PCA analysis of quality control samples in negative ion mode. **(C)** PLS-DA of differential metabolites between the model group and the blank group under positive ion mode. **(D)** PLS-DA of differential metabolites between the model group and the blank group under negative ion mode. **(E)** Volcano plot of the differential metabolites between the blank group and the model group under merge ion mode. **(F)** Volcano plot of the differential metabolites between the FAE group and the model group under merge ion mode. **(G)** Enrichment analysis of differential metabolite pathways between the FAE group and the model group. NOR, control group; MOD, model group; YZZ, high-dose FAE group.

The principal component analysis (PCA) results highlighted a clear separation between the model and blank groups, indicating distinct differences between the metabolites of mice with HF and those of the control group (Figures 2A,B). PLS-DA further revealed significant variations in metabolites between the model and blank groups, with notable disparities in both the positive- and negative-ion PLS-DA models (R2 = 0.996, Q2 = 0.934; R2 = 0.995, Q2 = 0.933, respectively) (Figures 2C,D). Based on the importance of VIP values, we used multivariate analysis along with the *p*-values and multiple differences for screening. Metabolites used for differential expression (Figure 2E). *p*-value <0.05, VIP ≥ 1, and fold change ≤0.833 or ≥ 1.2 (Tables 1, 2) were considered as the screening criteria. We integrated differential metabolites identified from mouse liver tissue, used pathway analysis modules to enrich the metabolic pathways involved in the aforementioned differential metabolites, and finally determined the metabolic pathway with the highest correlation with FAE intervention in HF based on impact >0.1 or *p* < 0.05 (Figures 2F,G). The most influential pathways were starch and sucrose metabolism, fructose and mannose metabolism, galactose metabolism, amino and nucleotide sugar metabolism, lysosomes, and taste transduction (Table 3).

**Table 1:** Top 20 differential metabolites between blank group and model group mice.

Compound	*p*	VIP	FC	Up/Down
3-methoxylimaprost	0	1.48	25.05	↑
Daidzein	0	1.52	0.04	↓
Avocadyne acetate	0	1.58	11.54	↑
cyano-3-[5-(2,5-dichlorophe3-nyll)-2-furanyl]-n-5-quinolinyl-2-propenamide	0	1.60	0.09	↓
[4-[(5,6-diphenyl-2-pyrazin3-yl)3-3-(1-methylethyl)amino]4-butoxy]4--Acetic acid	0	1.70	9.90	↑
Pro-Thr	0	1.66	0.10	↓
Pg 36:3	0	1.63	9.85	↑
Adipoyl-l-carnitine	0	1.69	9.54	↑
Ltc4-[d5]	0	1.34	8.89	↑
Sinefungin	0	1.40	0.11	↓
3-deacetylsalannin	0	1.64	0.12	↓
3-hydroxyphenazepam	0	1.60	8.25	↑
Notopterol	0	1.64	0.13	↓
1-stearoyl-2-docosahexaenoyl-sn-2-glycero-3-phospho-(1-sn-glycero)	0	1.7	7.56	↑
Acetylcitrulline	0	1.37	0.14	↓
5-methyltetrahydrofolic acid	0	1.26	0.14	↓
3-cyano-7-hydroxycoumarin	0	1.33	7.03	↑
3-6-[[3,4-dihydroxy-4-(hydroxymethyl)oxolan-2-yl]oxymethyl]-3,4,5-trihydroxyoxan2-yl]oxy-2-methylpyran-4-one	0	1.70	0.15	↓
Spiramycin	0	1.79	6.21	↑
Methyl deoxycholate	0	1.65	6.15	↑

**Table 2:** The top 20 differential metabolites between the FAE group and the model group of mice.

Compound	*p*	VIP	FC	Up/Down
Maltotetraose	0	2.43	0.11	↓
Stachyose	0	2.34	0.11	↓
D-turanose	0	2.27	0.12	↓
Fenamiphos sulfone	0	2.10	0.13	↓
Uroporphyrin i	0	2.14	0.14	↓
D-(+)-Galactose	0	2.20	0.16	↓
Melezitose	0	2.43	0.16	↓
1-(1,2r-dihexanoylphosphatidyl)inositol-3,5-bisphosphate	0	1.96	0.17	↓
Dihydrofolic acid	0	2.15	5.72	↑
Muramic acid	0	2.62	0.18	↓
Maltotriose	0	2.42	0.18	↓
Hematoporphyrin	0	1.99	5.17	↑
D-Threitol	0	2.42	0.20	↓
Laminaritetraose	0	2.29	0.20	↓
D-Mannose	0	2.33	0.21	↓
N-3-hydroxydecanoyl-l-homoserine lactone	0	1.66	0.22	↓
N-Formylmethionine	0	1.86	4.12	↑
3-galactosyllactose	0	2.33	0.25	↓
Sucrose	0	2.38	0.26	↓
Glutathione	0	1.88	0.26	↓

**Table 3:** Metabolic pathways involved in specific biomarkers.

Pathway	Total	Hits	*p*	Impact
Starch and sucrose metabolism	37	4	0.00	0.11
Fructose and mannose metabolism	55	5	0.00	0.09
Galactose metabolism	46	5	0.00	0.11
Amino sugar and nucleotide sugar metabolism	118	4	0.03	0.03
Lysosome	4	1	0.04	0.25
Taste transduction	33	3	0.00	0.09

### 3.4 Effect of FAE on the gut microbiome of mice with CCl_4_-induced HF

**Figure 3:**
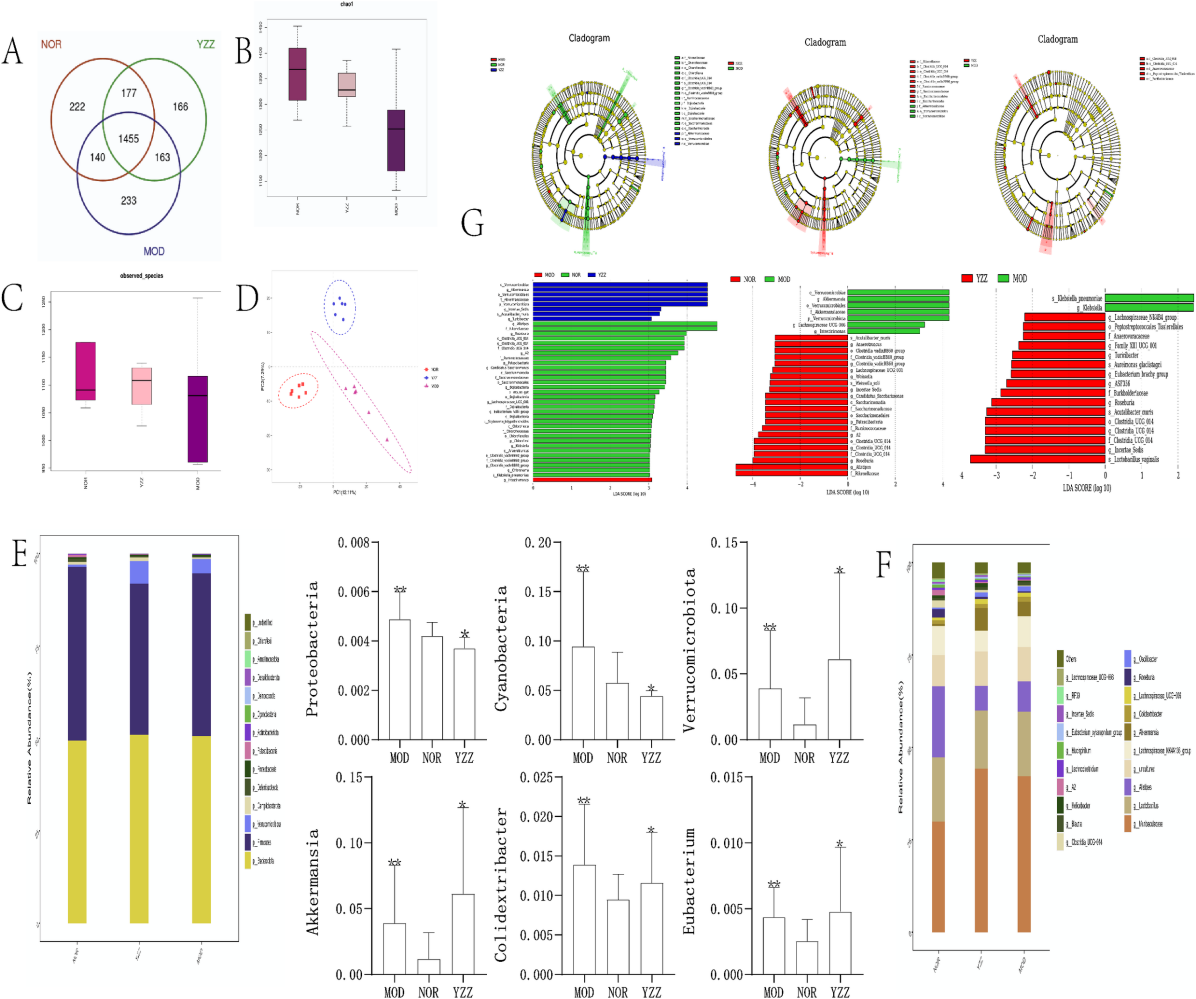
The effect of FAE on gut microbiota in mice with hepatic fibrosis. **(A)** OTU expression in each group. **(B)** chao1 indexes. **(C)** Observed species index. **(D)** PLS-DA diagrams. **(G)** Linear discriminant analysis effect size analysis. **(E,F)** Relative abundance of gut microbiota in each group at the phylum level. *n* = 8, ** *p* ≤ 0.05 versus NOR; * *p* ≤ 0.05 versus MOD. NOR, control group; MOD, model group; YZZ, high dose FAE group.

IlluminaPE250 sequencing of 16S rDNA genes from 24 fecal samples and subsequent clustering into operational taxonomic units (OTUs) identified 1903 OTUs. The sequencing coverage indices (> 0.99) indicated reliable data quality for all the three sample groups (Figure 3A). The dilution curves demonstrated that the sequencing data for the gut flora across the three sample groups were reasonable (Figure 3B).

Alpha diversity analysis of the gut microbiota revealed that the Chao1 index and observed species index were positively correlated with species richness, indicating increased diversity post-CCl_4_ induction but decreased diversity following FAE intervention (Figures 3B,C). The results of PLS-DA (Figure 3D) displayed a distinct separation among the three sample groups, emphasizing the significant differences in gut microbiota composition.

At the phylum level (Figure 3E), distinct dominant species, including *Firmicutes*, *Bacteroidetes*, and *Verrucomicrobiota*, were identified in the intestinal colonies of mice within each group. The relative abundance of *Firmicutes* and *Bacteroidetes* was the highest, with variations among the groups. *Cyanobacteria*, *Proteobacteria*, and *Verrucomicrobiota* were significantly enriched in the model group compared to those in the blank group. Moreover, the relative abundance of these phyla in the FAE group indicated their potential therapeutic effects on the modulation of gut microbiota composition.

At the genus level (Figure 3F), the relatively high abundance in each group of mice belonged to *Muribaculaceae* (*Muri*), *Lactobacillus*, and Alistipes (*Alis*), with *Muri* and *Lactobacillus* exhibiting the highest proportions. The relative abundance of *Muri* in each group was as follows: blank group: 29.96%; model group: 42.27%; FAE group: 42.23%. The relative abundance of *Lactobacillus* spp. was as follows: blank group: 17.34%; model group: 17.44%; FAE group: 15.74%. *Akkermansia*, *Colidextribacter*, and *Eubacterium* were significantly enriched in the model group compared to those in the control group (*p* < 0.05). Additionally, the relative abundance of *Akkermansia*, *Colidextribacter*, and *Eubacterium* was higher in the FAE group than in the model group (*p* < 0.05).

Upon setting the linear discriminant analysis threshold at 3, the linear discriminant analysis effect size analysis (Figure 3G) revealed significant differences in species abundance in the gut microbiome of the mice across all groups. In the blank group, the notable differential flora included *Rikenellaceae*, *Ruminococcaceae*, and *Saccharimonadaceae*, whereas the model group showed *Intestinimonas* as the differential flora. The FAE group was enriched in *Verrucomicrobiae* and *Akkermansiaceae*. Notably, the model group displayed an increase in the relative abundance of *Trichosporonaceae* (*Lachnospiraceae*) and *Verrucomicrobiota* and a decrease in that of *Cyanobacteria* and *Proteobacteria* when compared with the blank group. The FAE group showed increased relative abundance of *Verrucomicrobiota* and *Akkermansia*. They exert an inhibitory effect on the progression of HF in mice.

### 3.5 Correlation between the gut microbiota and metabolites

**Figure 4:**
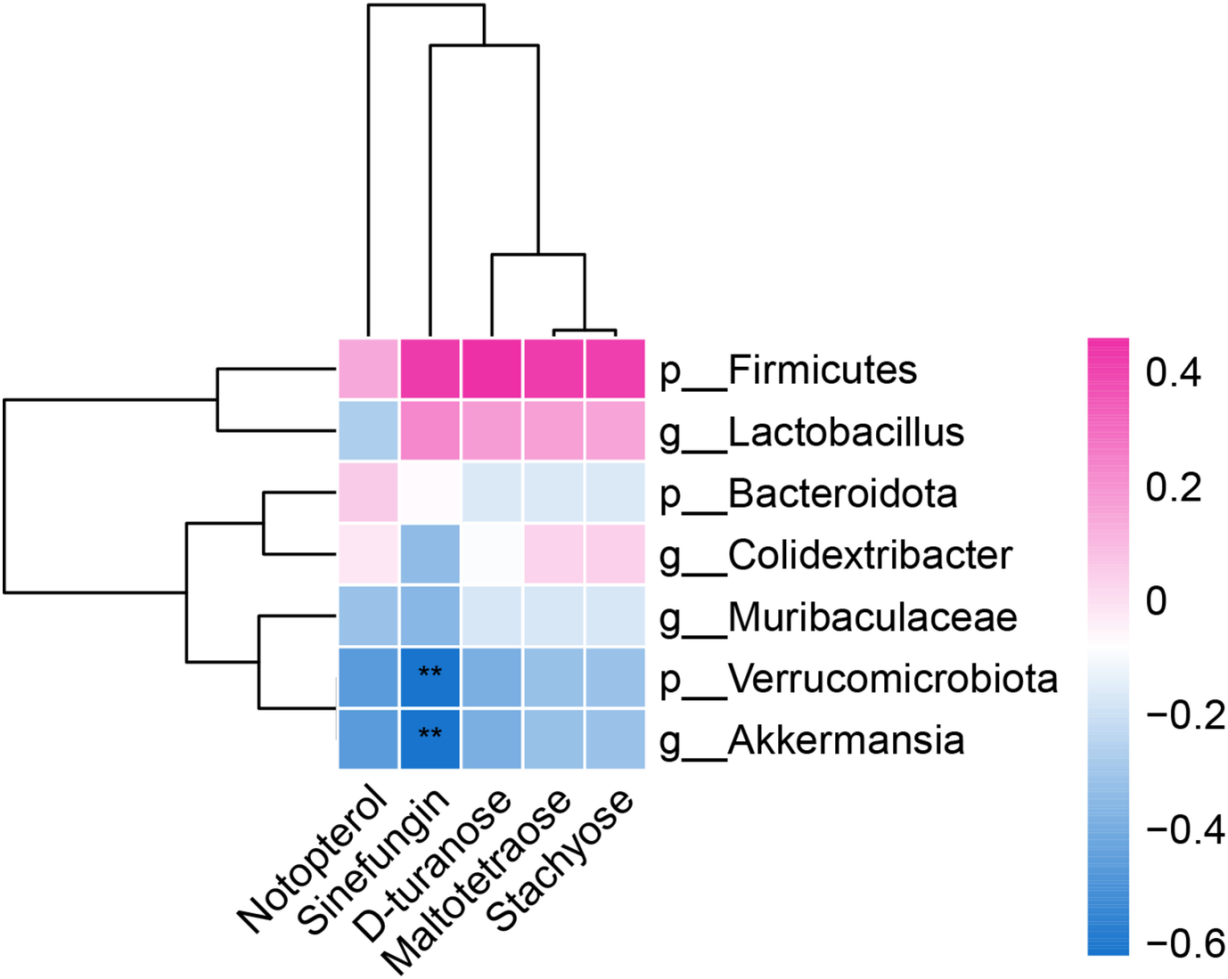
Correlation analysis heatmap between the level of gut microbiota and differential metabolites in the liver.

To further explore the relationship between the intestinal flora and metabolites, Spearman correlation analysis was utilized to correlate the mouse liver differential metabolites with the flora at the portal and genus levels and to generate correlation heatmaps (Figure 4). Positive and negative correlations are depicted in red and blue, respectively. Color intensity indicates the strength of the correlation coefficient.

The differential metabolites Notoperol and Sinefungin were downregulated in both the blank and model groups, with stronger downregulation observed in the model group. They exhibited negative correlations with the differential genera *Verrucomicrobiota*, *Akkermansia*, and *Muribaculaceae*. Additionally, Sinefungin negatively correlated with the differential genus *Lactobacillus* and positively correlated with the genus *Firmicutes.*

Similarly, the differential metabolites D-turanose, Maltotetraose, and Stachyose were downregulated in both the model and FAE groups. They showed negative correlations with the genera *Verrucomicrobiota* and *Akkermansia* but positive correlations with the genera *Firmicutes*, *Lactobacillus*, and *Colidextribacter*. These findings provide insights into the intricate relationship between the intestinal flora and metabolites.

### 3.6 Drug target identification and network construction

**Figure 5:**
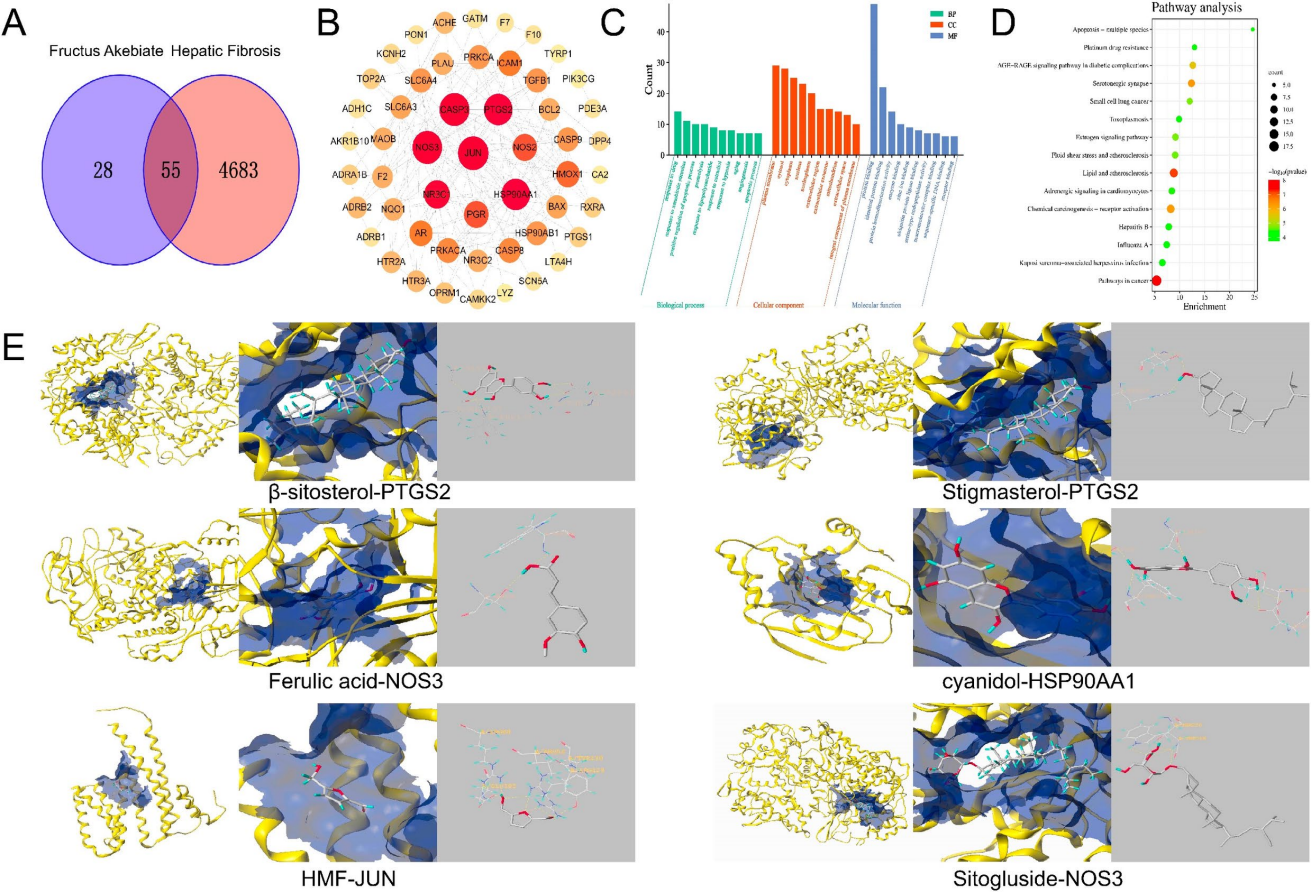
Network pharmacological analysis chart. **(A)** Venn diagram of the intersection between component targets and disease targets. **(B)** PPI network. **(C)** GO enrichment analysis results. **(D)** Bubble diagram of the anti fibrotic pathway of FAE. **(E)** The molecular docking pattern with the highest total score.

NP analysis was conducted to explore the anti-HF mechanism of the FAE. Initially, 83 drug targets and 4,738 HF-related targets were identified using the Uni-Prot prediction database. The intersection of these sets was visualized using a Venn diagram (Figure 5A). Utilizing the network analyzer tool in Cytoscape 3.7.2 software, a network topology analysis was performed on the interaction network diagram of “traditional Chinese medicine-active ingredient-intersecting target.” The mean values of the meso-, proximity centrality, and degree of network topology parameters were 0.057, 0.39, and 6.23, respectively, and the active ingredients with median, proximity centrality, and degree values exceeding the average were identified as FAE core active ingredients (Table 4).

**Table 4:** Topological parameters of core active component network in Akebiae Fructus.

Designation	The mean values of meso	Proximity to centrality	Degree
β-sitosterol	0.358319	0.48125	25
Stigmasterol	0.096531	0.41847826	13
Ferulic acid	0.108039	0.41397849	12
cyanidol	0.072798	0.39285714	7
HMF	0.130253	0.39690722	8
Sitogluside	0.106496	0.40957447	11

A protein–protein interaction (PPI) network, consisting of 52 nodes representing functional proteins and 189 edges representing the interactions between functional and other proteins, was constructed using Cytoscape (Figure 5B). Within the network, 11 key targets, including JUN, CASP3, NOS3, PTGS2, HSP90AA1, NR3C1, PGR, NOS2, HMOX1, PRKACA, and SLC6A4, which were determined based on node degree, were identified (Table 5). These key targets may play crucial roles as target genes in the treatment of HF. Furthermore, to analyze the mechanism of action of FAE in HF treatment, 55 intersecting targets were subjected to GO function enrichment analysis in the DAVID database, with a significance threshold set at *p* < 0.05. A total of 207 biological process (BP) pathways, 36 cellular component (CC) pathways, and 55 molecular functions (MF) were enriched in the intersecting targets. The top ten pathways for BP, CC, and MF were selected and presented in a drift diagram (Figure 5C).

**Table 5:** Network topology parameters of key target of Akebiae Fructus.

Target	The mean values of meso	Proximity to centrality	Degree
JUN	0.136334	0.600000	22
CASP3	0.112194	0.579545	21
NOS3	0.209135	0.586207	21
PTGS2	0.059845	0.560440	19
HSP90AA1	0.084829	0.579545	19
NR3C1	0.098259	0.536842	15
PGR	0.065055	0.515152	13
NOS2	0.069383	0.510000	13
HMOX1	0.008379	0.495146	12
PRKACA	0.111631	0.504951	10
SLC6A4	0.028562	0.428571	9

The 55 intersecting targets were also analyzed for KEGG pathway enrichment in the DAVID database, resulting in a total of 85 enriched KEGG pathways. The top 15 KEGG pathways with the smallest *p*-values were selected to create a KEGG pathway bubble diagram (Figure 5D). The targets involved in anti-HF prediction were associated with various signaling pathways, including pathways involved in cancer, lipid and atherosclerosis, serotonergic synapses, chemical carcinogenesis-receptor activation, AGE-RAGE signaling pathway in diabetic complications, and the estrogen signaling pathway.

Overall, these findings shed light on potential mechanisms underlying HF treatment and highlight the significance of key target genes in HF therapy.

**Figure 6:**
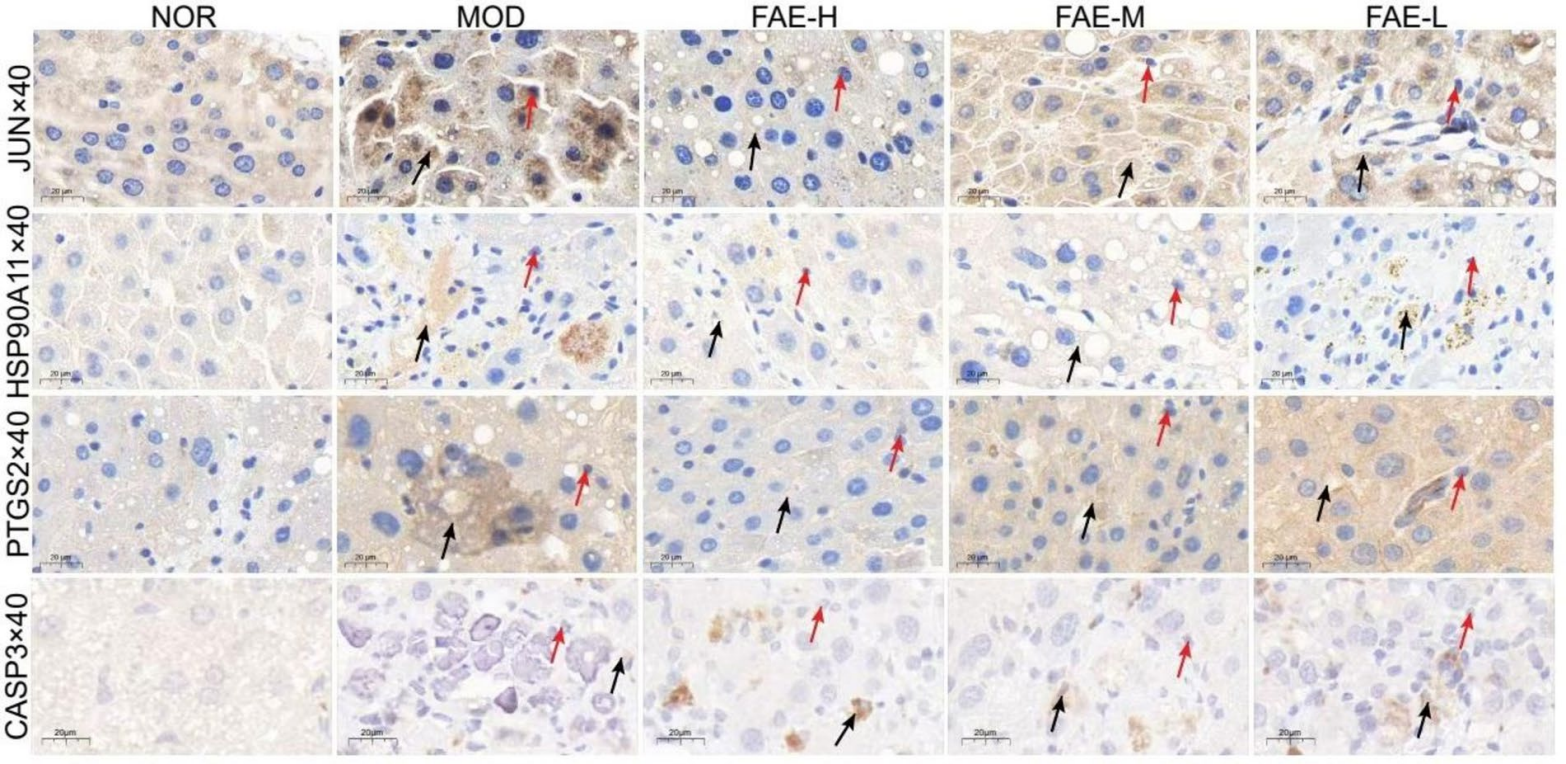
Immunohistochemistry of JUN, HSP90A11, PTGS2 and CASP3. →: necrocytosis; →: the express of JUN, HSP90A11, PTGS2, CASP3. NOR, control group; MOD, model group; FAE-H, high-dose FAE group; FAE-M, medium-dose FAE group; FAE-L, low-dose FAE group.

Six key targets, JUN, CASP3, NOS3, PTGS2, HSP90AA1, and NR3C1, were selected for molecular docking with *β*-sitosterol, stigmasterol, ferulic acid, carotenoid, anthocyanin, and 5-hydroxymethylfurfural, which are the core active components in the treatment of HF. Among these key targets, the highest binding activities were observed for HSP90AA1, PTGS2, NOS3, HSP90AA1, JUN, NOS3. The core active components HSP90AA1, PTGS2 and NOS3 exhibited favorable binding affinities for the aforementioned six targets (Figure 5E). JUN, CASP3, NOS3, PTGS2, HSP90AA1, and NR3C1 have good binding activity with the core components of treating HF, suggesting that these may be the core targets for intervening in HF. Through immunohistochemical analysis of the levels of JUN, HSP90AA, PTGS2, and CASP3, it was found that compared to the blank group, the JUN, PTGS2, and HSP90AA levels in the model group were significantly increased, while the FAE intervention group showed a significant decrease, and the CASP3 content was reduced in the model group and significantly increased in the FAE intervention group (Figure 6).

## 4 Discussion

HF, a physiological and pathological process of abnormal hyperplasia of the hepatic connective tissue caused by sustained liver injury, is a common pathological process in various liver injuries (19). Late-stage HF can develop into cirrhosis, and the only available curative treatment option for patients with advanced cirrhosis is liver transplantation. Although no drugs have been developed to completely cure HF, studies have demonstrated that early HF can be reversed through drug intervention; therefore, it is important to take active and effective measures in the early stages of HF to prevent further deterioration of the condition (20). Therefore, developing efficient, low-toxicity, multi-target for HF is necessary. We combined intestinal microbiome analysis and metabolomics to explore the pathogenesis of HF (19).

The physiological function of the liver and spleen is related to the intestinal microbiota, and dysbiosis is known to affect the pathological state of liver depression and spleen deficiency syndrome in traditional Chinese medicine (21). Metabolomics research is currently widely conducted in various fields of basic and clinical research, and alterations in metabolic pathways have a considerable impact on the progression of HF in mice (22). Traditional Chinese Medicine believes that FAE belongs to the liver and spleen meridians, and has the effects of soothing the liver, regulating qi, promoting blood circulation, relieving pain, and relieving irritability and diuresis (17). In modern pharmacology, FAE has also been confirmed to regulate metabolism and anti-inflammatory effects, and studies have reported that precursors can be used in the treatment of hepatic fibrosis and liver cancer (23). Liver damage due to metabolic imbalance, immune or viral invasion can form hepatic fibrosis, and eventually develop cirrhosis or liver cancer. When the liver is damaged, overexpression of inflammatory factors in the body can lead to a significant increase in inflammatory cells. FAE has a good anti-inflammatory effect and can help the body inhibit the further development of inflammation; FAE can also reduce the levels of TG in the blood, promote fat breakdown and metabolism, and help inhibit the further development of hepatic fibrosis. Therefore, we speculated that FAE has the role of promoting liver metabolism and anti-inflammatory.

Ultra-performance liquid chromatography-mass spectrometry-based techniques revealed that FAE significantly altered hepatic metabolites in mice with HF. In the CCl_4_-induced HF mouse model, glutathione, cytosolic acid, anilinophosphosulfone, and kombucha tetrasaccharide were upregulated, whereas dihydrofolate, hematoporphyrin, and N-formylmethanethionine were downregulated in the prenylated group. The components of starch and sucrose metabolism, fructose and mannose metabolism, galactose metabolism, aminoglycan and nucleotide glycoside metabolism, lysosomal enzymes, and taste transduction pathways were significantly altered. Cytidylic acid has a significant cough suppressant effect. Lipopolysaccharide can alter the cellular metabolism of the body, cause a variety of non-specific immune responses, and inhibit the growth and reproduction of various microorganisms such as bacteria and viruses. Glutathione serves many pivotal functions in the central nervous system, including the modulation of cellular differentiation and proliferation, apoptosis, enzyme activation, metal transport in cells, neurotransmission, and as a source of cysteine during protein synthesis (24). FAE may use elevated levels of glutathione and cytarabine to improve inflammation and thus intervene in HF.

To further explore the mechanism underlying the effect of prebiotics on HF, we used 16S rDNA gene sequencing to detect alterations in the intestinal flora of mice. The liver anatomically belongs to the same digestive system and is closely connected to the intestine. The formation of a mutually beneficial symbiotic homeostatic environment between intestinal microorganisms and the organism is important for liver health. Ecological imbalances and disorders of intestinal microflora can lead to damage to the intestinal barrier and intestinal inflammation, which in turn can lead to intestinal mucosal damage (25). Chinese medicine can improve chronic liver disease through intestinal flora (26–28). We found that the traditional Chinese medicine FAE had a substantial influence on the diversity of intestinal flora in mice, and the number of OTUs in the FAE group differed greatly from that in the other two groups. The Chao1 index increased significantly, and the PLS-DA showed that the three groups of samples were far away from each other; additionally, the species differed considerably. The analysis of changes in the intestinal flora and species composition revealed that prebiotics significantly increased the abundance of *Verrucomicrobiota*, *Akkermansia*, *Colidextribacter*, and *Lactobacillus* and reduced that of *Cyanobacteria*, *Proteobacteria*, and other bacterial groups in HF mice, which is consistent with the results of previous studies (29, 30). Research has found that probiotics such as *Verrucomicrobiota* and *Akkermansia*, Colidextribacter, Lactobacillus bacteria work by protecting the intestinal barrier, participating in maintaining intestinal immune homeostasis, and reducing liver inflammatory cells, while harmful bacteria such as Proteobacteria increase the risk of inflammation; This is consistent with our experimental results (31). Therefore, it was hypothesized that prebiotics can exert a protective effect on HF in mice by increasing the abundance of certain intestinal flora, such as *Akkermansia* and *Verrucomicrobiota*, and by altering the levels of metabolites such as glutathione, cytidylic acid, anilinophosphosulfone, and kombucha tetrasaccharides.

The correlation analysis between the key metabolites and the different intestinal flora showed that Notoperol and Sinefungin exhibited negative correlations with the differential genera *Verrucomicrobiota*, *Akkermansia*, and Muribaculaceae. Sinefungin negatively correlated with the differential genus Lactobacillus and positively correlated with the genus Firmicutes. D-turanose, Maltotetraose, and Stachyose showed negative correlations with the genera *Verrucomicrobiota* and *Akkermansia* but positive correlations with the genera Firmicutes, Lactobacillus, and Colidextribacter. These key metabolites are highly correlated with the gut microbiota. From this, we can conclude that the above key metabolites are highly correlated with gut microbiota, indicating that FAE may reverse the progression of hepatic fibrosis by regulating gut microbiota through the gut liver axis and affecting liver metabolism. Its specific mechanism of action needs further research and verification.

NP was used to screen the core active ingredients and main pathways of action of FAE. The binding activities of the core active ingredients and key targets were verified through molecular docking; HSP90AA1, PTGS2 and NOS3 were found to have the highest binding affinities for the key targets. In recent years, several experimental studies have demonstrated that these core active ingredients, targets, and pathways play important roles in the treatment of HF. Tingyu Ban et al. found that flavonoids could regulate cancer pathways by controlling targets such as PTGS2, HSP90AA1, PGR, and PRKACA, thus treating HF caused by non-alcoholic fatty liver disease (32). The active ingredient PTGS2 acts on HSP90AA1 to inhibit renal fibrosis through the AGE-RAGE signaling pathway (33). Organic acid components can reduce the expression of caspase 3 and inhibit hepatocyte apoptosis, thereby reducing the risk of HF (34). The anthocyanins in prebiotics belong to flavonoids, and they can significantly reduce the expression of Bcl-2 protein, promote the release of cleaved caspase-3, and prevent HF caused by alcoholic liver disease. Ezhilarasan Devaraj et al. verified that *β*-sitosterol, a steroidal component of prebiotics, can reduce the relative expression of SOD and CAT and alleviate the risk of HF. Ferulic acid, a constituent of the organic acids in prepared seeds, can reduce the activities of serum hepatic enzymes and ameliorate HF by directly binding to and inhibiting the expression of PTP1B. JUN can prevent excessive activation of endoplasmic reticulum stress response in liver cells, thereby reducing liver cell damage and apoptosis (35). HSP90AA1 promotes the activation and expression of hepatic stellate cells. PTGS2 is poorly expressed in resting cells and most tissues in normal physiological conditions, but its expression usually increases dramatically within 24 h when the tissue or cell is stimulated to cause pain or inflammatory response (36). CASP3 can promote cell apoptosis. We speculate that FAE can further regulate signaling pathways closely related to HF by regulating the expression of, JUN, HSP90AA1 PTGS2, CASP3 etc. The specific mechanism needs further experimental elucidation.

In summary, prebiotics can inhibit the progression of HF by increasing the abundance of beneficial bacteria and reducing that of harmful bacteria in mice with HF, thereby altering the metabolic pathway. However, the targets, signaling pathways, and mechanisms whereby prebiotics work are still unclear and require further investigation.

## 5 Conclusion

In this study, 16S rDNA high-throughput sequencing was used to determine FAE could significantly increase the abundance of *Akkermansia* and *Verrucomicrobiota* to resist HF. Using liver metabolomics, we found that FAE could reverse HF by regulating the changes in some metabolites. Moreover, NP detected six targets for the treatment of HF, including the JUN/CASP3/NOS3/PTGS2/HSP90AA1/NR3C1, and identified potential targets and pathways. Taken together, we speculate that some active components in the prediction can treat HF, represent effective targets for the treatment of HF, and provide a basis for the development of FAE.

## Data Availability

All relevant data is contained within the article: The original contributions presented in the study are included in the article/Supplementary material, further inquiries can be directed to the corresponding author.

## Footnotes

^1^ http://tcmspw.com/tcmsp.php

^2^ https://bioinfogp.cnb.csic.es/tools/venny/index.html

^3^ http://string-db.org

^4^ http://www.rcsb.org
